# Health care provision for refugees in Germany – one-year evaluation of an outpatient clinic in an urban emergency accommodation

**DOI:** 10.1186/s12913-018-3174-y

**Published:** 2018-06-25

**Authors:** Hannah S. Borgschulte, Gerhard A. Wiesmüller, Anne Bunte, Florian Neuhann

**Affiliations:** 1 Public Health Department Cologne, Cologne, Germany; 2 Institute of Occupational Medicine and Social Medicine, University Hospital RWTH Aachen, Aachen, Germany; 3 Institute of Public Health, University Hospital Heidelberg, Heidelberg, Germany

**Keywords:** Refugees, Health care, Emergency accommodation, Low-threshold access, Germany, Cologne, 2015

## Abstract

**Background:**

In 2015, Germany recorded the highest rates of refugees since the early 1990s. Access to medical care is a legally regulated fundamental element of aid for refugees. In practice, there are several hurdles such as language barriers and legal regulations. In response to the massively increased need, special outpatient services for refugees were started in several German cities. In Cologne, an outpatient clinic (OPD) was established in the largest emergency accommodation centre for refugees supported by the Cologne municipality and operated by the German Red Cross and physicians from the Association of Statutory Health Insurance Physicians. This study reports experiences of the first year of the OPD regarding structure, processes and utilization.

**Methods:**

Employing mixed methods, between May and December 2015 cross sectional pseudonymized data from patients’ contacts were collected, coded in the International Classification of Primary Care (ICPC) and evaluated. Infrastructure, equipment, process organisation and function of the OPD were assessed during five participatory observations and triangulated with results of a self-administered questionnaire for staff and four qualitative interviews with key informants.

**Results:**

During the observation period a total of 2205 persons (67% male) stayed in the emergency accommodation and 984 patient contacts (51% male) were registered, mainly by young persons from Western Balkan countries and Syria. Medical treatment was sought primarily for acute respiratory-, loco-motor-system- and skin symptoms followed by chronic physical diseases. Headache, back and neck pain and acute respiratory infection were the most frequent diagnoses. Questionnaires and interviews among staff revealed language barriers and psycho-trauma as the most frequently reported challenges. Equipment and staffing was adequate, but patient documentation was not systematic, leading to loss of information.

**Conclusion:**

To facilitate refugees’ appropriate access to health care, the OPD was seen as functional for this refugee accommodation centre. Need was recognised for standardized, data protective documentation and a health passport for clients for medical information. Psychological support for refugees needs expansion taking legal circumstances and coverage of costs into consideration. To improve patient communication employees working with refugees should be offered an introduction to culturally sensitive understanding of health and illness.

**Electronic supplementary material:**

The online version of this article (10.1186/s12913-018-3174-y) contains supplementary material, which is available to authorized users.

## Background

In 2015, the UN Refugee Agency (UNHCR) estimated that there were 65.3 million forcibly displaced people worldwide [1]. The civil war in Syria has forced an estimated 4.9 million people to leave their home country and this has resulted in the largest number of refugees^1^ up to 2015. Most of the refugees have sought shelter in bordering countries, but in recent years more and more refugees have come to Europe, and the Federal Republic of Germany (FRG) has become an important destination [1].

Reasons to be granted asylum in FRG are political persecution, which is statutorily addressed in § 16 of the German Constitution, and persecution *“for reasons of race, religion, nationality, membership of a particular social group or political opinion”* according to the Convention Relating to the Status of Refugees (1951 Refugee Convention) [2, 3].

Access to health care for asylum seekers in the FRG is regulated by the Asylum-Seekers’ Benefits Act, which restricts social services for asylum seekers. Access to health care focusses on *“acute diseases and pain”* [4, 5]. Treatment for chronic diseases requires approval by the social security office of the receiving municipality paying for medical services. This is often criticized because chronic diseases such as diabetes mellitus type 2 can acutely deteriorate. In these cases, the need of the approval process can lead to delays in treatment. Furthermore, language barriers and poor knowledge of the health system restrict refugees’ access to health care [6] thus, refugees can be seen as vulnerable persons with regard to health care [7].

**Fig. 1:**
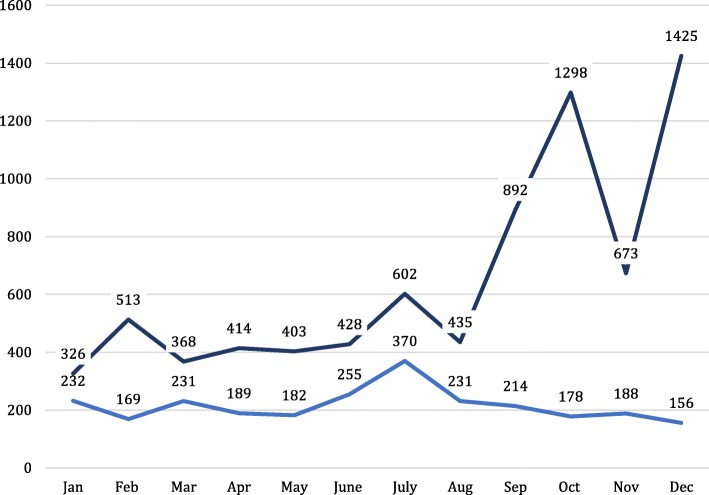
Development of the numbers of arriving refugees in Cologne in 2015. The term “officially allocated” means refugees registered in initial registration offices and then allocated to the emergency accommodation in Cologne. “Independent arrivals” refers to refugees directly arriving in the emergency accommodation in Cologne. officially allocated. Independent arrivals.  officially allocated,  independent arrivals

In 2015, Cologne received and accommodated a weekly average of 150 refugees (Fig. 1) reaching approximately 1000 per month in the last quarter of that year. Accommodation facilities were set up by city authorities in former office buildings, vacant large stores and sports halls and managed by third parties like the German Red Cross (GRC). In response to the refugees’ increasing needs and calls for emergency care, the city authority with the GRC and the Association of Statutory Health Insurance Physicians (ASHIP) set up an outpatient clinic (OPD) at the largest emergency accommodation centre. The centre has a bed capacity of 558 and was housing on monthly average 730 refugees.

We describe and analyse the OPD set-up, usage and experiences for the study period from May to December 2015. Data about the refugees seeking consultation, reasons for consultation, complaints and results of the interviews of the OPD employees are presented here. Additionally, data on presented complaints were retrospectively retrieved from records of returning patients for the period January to end of April 2015 and added to the overall evaluation of health complaints.

## Methods

For the evaluation, quantitative and qualitative methods were applied.

Data on the number of inhabitants, their countries of origin, age and gender were obtained on a monthly base from the register of the institution. The total number of inhabitants included all persons who ever moved in until December 2015 subtracted by the number of persons who moved out before May 2015. Data on the utilization of the OPD was extracted from the clinic register and all were entered in a Microsoft Excel® data file and compared with the total and monthly occupancy of the institution for the period May to December 2015. Pseudonymized data of the assessed patients were captured from doctors’ documentation, coded in the International Classification of Primary Care (ICPC) and evaluated retrospectively [8]. Written documentation on patients was only kept at the OPD from May 2015 onwards. Prior to that, only the patients received some medical notes. All registered data from May to end of December 2015 was included in the analysis. An analysis of patient contacts between January and end of April 2015 was only possible if the patient visited the OPD again after May 2015. These results were included in the analysis of the frequent complaints. Demographic data of the inhabitants and patients were compared to data of the accommodation inhabitants in general with regards to country of origin, age and gender. Only descriptive statistically methods were applied using Microsoft Excel®.

The patient data on symptoms and complaints from the ICPC were further categorized in “acute somatic”, “chronic somatic”, “suspected mental health related”, “need for vaccination”, “pregnancy related”, “request for medical certificate” and “unspecific” to get an overview for the different categories for request.

Secondly, there were five participatory observations capturing the structure and processes of the accommodation centre’s OPD department by the one of the researchers. Finally, views and experiences of the doctors (*n* = 17), the two nurses and the social worker responsible for the facility were captured using a self-administered questionnaire in January 2016 send as read-only word document via email (*n* = 20, response = 16). Next to basic personal information the participants were asked on personal and medical equipment and the organisation of the OPD, documentation, interaction among stakeholders, interaction and communication with the patients, perceptions of reasons for consultations. Open and multiple selection questions were used. To answer the open questions, a scale ranging from 0 to 10 was used. An answer could be given marking a value on the scale between “I disagree” (0) and “I totally agree” (10) or “very bad” (0) and “very good” (Additional file 1) (10). In addition, key informant interviews were conducted with purposively selected staff: two doctors representing general medicine and paediatrics and having the highest number of working hours in the OPD, one of the 2 nurses and the responsible social worker (*n* = 4) focussing on the topics of *documentation* and *difficulties in patient contact.* Data from the questionnaire was likewise entered in a spreadsheet and descriptively analysed. The 4 interviews were audio recorded and the researcher took handwritten notes. Recordings and key remarks were thematically analysed along the same topics as for the questionnaires. For the overall analysis, the results of the different methods were triangulated.

## Results

### Setting

**Fig. 2:**
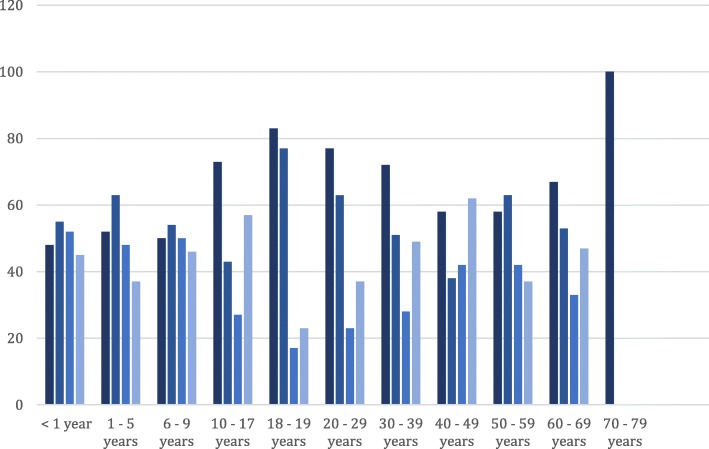
Distribution of the inhabitants and patients with regard to gender and age on average in the period between May and December 2015 based on data of 2.169 of 2.205 inhabitants and 964 of 984 patient contacts.  % male inhabitants,  % male patient contacts,  % female inhabitants,  % female patient contacts

**Fig. 3:**
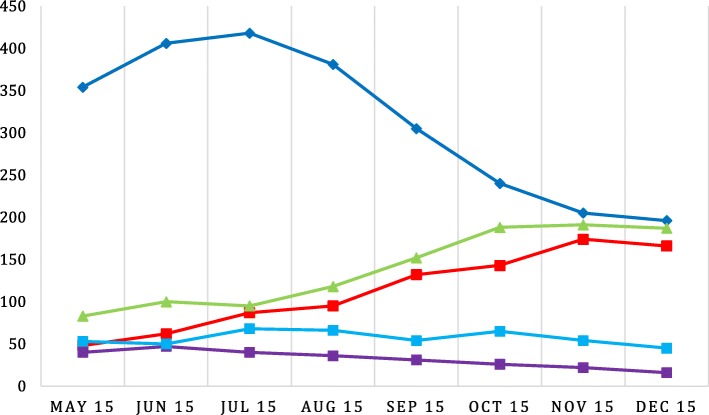
Distribution of the inhabitants of all ages with regard to their origin. Western Balkan includes the countries Albania, Bosnia, Kosovo, Macedonia, Montenegro, and Serbia. Middle East includes the countries Afghanistan, Bangladesh, Lebanon, India, Iraq, Iran Palestine, and Pakistan. North Africa includes the countries Algeria, Egypt and Morocco. Africa includes the countries Angola, Congo, Eritrea, Gabon, Ghana, Guinea, Mali, Nigeria, Senegal, Sierra Leona, and Somalia (*n* = 2.2055,; missing: 178).  Western Balkan,  Syria,  Middle East,  North Africa,  Africa excl. North Africa

The OPD described here was set up in the largest emergency accommodation for refugees in Cologne, a former administrative office building. The occupancy of the accommodation changed daily due to newly accommodated persons and people moving out. During the observation period May to December 2015 a total of 2205 persons were registered in the emergency accommodation. Officially there were 558 beds but the average occupancy rate was 730 per month. Rooms accommodated between 2 and 6 people. Full data was available for 2169 out of 2205 persons. The inhabitants were mainly young and male (Fig. 2). A total of 71% of the inhabitants were less than 30 years old and 67% of the whole population were male. In the adult population, 73% were male and even 81% among the 18–19 years old. The inhabitants’ countries of origin changed throughout the observation period (Fig. 3). In May, 58% of the refugees were from Western Balkan^2^ countries, which decreased to 29% in December. In the same period, the percentage of persons from Syria increased from 8 to 25%.

Starting in January 2015, the OPD was operated by the GRC and physicians from ASHIP. The outpatient clinic intended to facilitate access to health care for the tenants in the emergency accommodation without establishing a parallel structure to the regular health care system. The outpatient clinic offered basic medical care and was a first health contact point for the refugees.

Before the OPD was introduced, the Cologne medical rescue service was frequently called by tenants and staff. In January 2015, the clinic was set up in a purposively equipped container and opened twice a week for adults (Tuesday from 9 to 12 a.m. and Wednesday from 2 to 5 p.m.) and once a week for children (Wednesday from 2 to 5 p.m.). One doctor and a nurse were responsible for one appropriate consultation. Tenant access was open during consultation hours. Every contact was registered by the nurses (patient name, date of birth and nationality). A brief medical history, medical procedures and therapy suggestions were recorded by the doctors at their discretion in a paper folder remaining with the patient. The OPD equipment included an examination bed, a manometer, a clinical thermometer, a weighing scale, dipsticks for urine tests and a blood glucometer. Bandaging material and some drugs such as antibiotics, ibuprofen, diclofenac and drugs for scabies were stored in the OPD. Since a regular translation service was not available during consultation hours, web-based translation programs, multilingual staff members or peers with basic knowledge of German assisted. Medically indicated referral to a regulatory health care system was possible for both hospital and ambulatory services. The costs for medical services were paid by the Cologne’s social security office.

### OPD user profile

Overall in 2015, the service registered 1692 contacts by adults and children. In the observation period from May to December 2015, 964 contacts were listed of which 669 were adults. Males represented 51% of adult’s consultations and 56% of the children’s consultations. Among the adults, the largest group of patients was between 20 and 29 years of age (Fig. 2). Persons from Western Balkan countries constituted the major group in both consultations (59% of adult patients). With fewer people arriving and being accommodated from that region their proportion among the users of the OPD declined as well. Children less than one year of age represented 20% of patients in the consultation hours for children. Most children were between one and five years old (46.8%). The vast majority (83%) of them came from Western Balkan countries. The share of children from this region in the paediatric clinic remained relative high throughout the year representing 100% of the visits in May 2015 and 45 and 63% in August and December respectively.

Compared to the total number of residents in the emergency accommodation centre, those accessing OPD services were more often women, older people and persons from Western Balkan countries.

Adding the data from a partial recording since January 2015 (includes information about patients who visited the consultation for two or more times after May 2015), 828 contacts were recorded by 422 patients. However, more than half (274/422) of the patients visited the consultation only once.

### Reasons for consultation

**Fig. 4:**
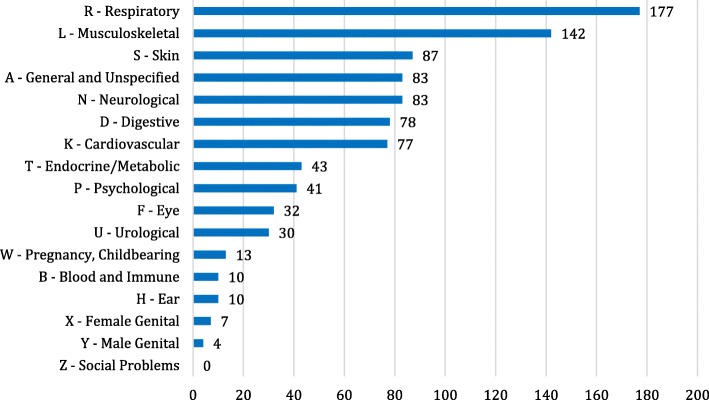
Distribution of diagnoses arranged by organic system in the International Classification of Primary Care (ICPC) including all patient contacts during consultation hours for adults between May and December 2015 (*n* = 917; missing: 13)

**Fig. 5:**
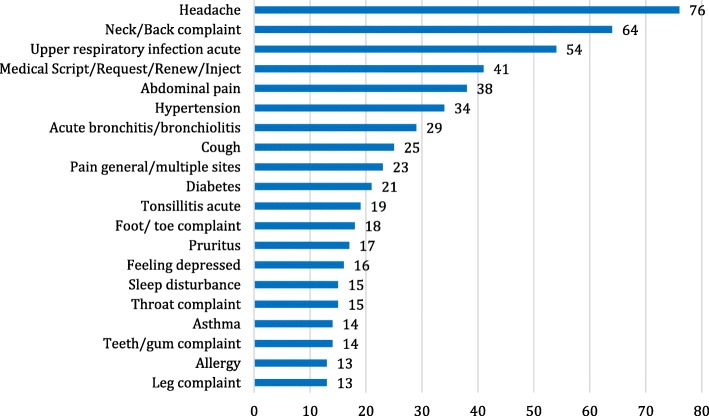
Representation of the 20 most frequent diagnoses (number: 559) during consultation hours for adults between January and December 2015 (*n* = 1.073; missing: 21)

Reasons for adult OPD visits were mainly acute physical illness (65%) and chronic diseases (28%). Respiratory tract complaints (19%) were most frequent, followed by complaints concerning the loco-motor apparatus (15%), the neurologic system (9.1%), circulatory system (8%), digestive system (8%) and skin (9.5%) (Fig. 4). Headache (7.1%), back pain and neck pain (6%), as well as acute infections of the upper respiratory tracts (5%) after ICPC were most frequently mentioned (Fig. 5). The remaining 7% concerned mental problems, pregnancies, medical expert reports and vaccinations. In 52% of the cases, drug therapy was initiated or continued. If medically appropriate, the patient was generally referred to dermatology (10.4%), ophthalmology (9.2%), otolaryngology (ENT) (9.2%), gynaecology (8.7%) or surgery (8.7%).

In addition, 83% of the children being admitted to the consultation suffered from acute physical complaints, mainly concerning respiratory organs, digestive system and skin (Table 1). The most frequent complaints were infections of the upper respiratory tract, cough and sneezing (Table 1). In 55% of the cases, a drug therapy was prescribed and the patient referred to ENT (19%) or ophthalmology (15%).

**Table 1:** Distribution of diagnoses arranged by organic system in the International Classification of Primary Care (ICPC) including all patient contacts during consultation hours for children between May and December 2015 (bold) (*n* = 379; missing: 6); supplemented by the most frequent diagnoses during consultation hours for children between January and December 2015 (*n* = 415; missing: 8)

A - General and Unspecified	51	13,5%
Congenital Anomaly	6	1,6%
Fever	21	5,5%
Medical Exam	15	4,0%
B - Blood and Immune	3	0,8%
D – Digestive	39	10,3%
Gastrointestinal infection	13	3,4%
F – Eye	20	5,3%
Conjunctivitis infectious	10	2,6%
H – Ear	18	4,7%
Acute otitis media	12	3,2%
K – Cardiovascular	4	1,1%
L – Musculoskeletal	12	3,1%
N – Neurological	4	1,1%
P – Psychological	2	0,5%
R – Respiratory	174	46,0%
Acute bronchitis/bronchiolitis	14	3,7%
Cough	28	7,4%
Sneezing	25	6,6%
Tonsillitis acute	16	4,2%
Upper respiratory infection acute	93	24,5%
S – Skin	42	11,1%
T – Endocrine/Metabolic	1	0,3%
U – Urological	2	0,5%
W – Pregnancy, Childbearing	1	0,3%
X – Female Genital	1	0,3%
Y – Male Genital	5	1,3%
Z – Social Problems	0	0%

### Results of staff questionnaire and participatory observation

Sixteen out of 20 staff completed the questionnaire survey. Among the respondents there were two nurses (female), one social worker (female), six family doctors (four female, two male) and seven paediatricians (six female, one male) with an age range between 29 and 64 years. Five of the staff members had a migratory background. Most of the doctors worked between 5 and 10 days in the outpatient clinic during the observation period. Doctors performed the work in the outpatient clinic next to their general work in their own practise and received refunding at the same rate as in the general practise through the social service. With regards to the operating hours, 75% of the respondents considered the opening hours per week for the adults and children as *“exactly sufficient”.* During the consultation hours, treatments were documented in paper-based records that clients were supposed to bring along for any medical consultation. Sixty-nine percent of the health care staff rated this system “*useful*”. However, when asked whether “*the documentation system leads to loss of information*”, the participants on the scale from 1(no loss) -10 (complete loss) rated it on average 4.7. When recording the data from the existing documentation system for the evaluation of the patients’ contacts, there were repeated losses because the documentation was non-standardized, handwritten and sometimes illegible. Almost all respondents mentioned *“language barriers”* followed by *“trauma”*, *“level of education”* and the *“social situation”* as barriers during patient contact. The assessment of psychological symptomatology as the reason for the patient’s visit in the qualitative interviews and questionnaires was rated higher than the number of the actual diagnoses in this category. In the qualitative interviews, this circumstance was explained by the fact that a psychological burden of the patient was often informally “*noticed*” during the contact. However, language problems prevented staff from ascertaining the patient’s medical history and so the patients’ symptoms were rarely translated into a diagnosis. This is illustrated by the following quote “*I can see this in the posture, facial expression and the ability of the patient to communicate.*” […] *“or in recurring occurrence of pain symptoms*” (Interview with female family doctor in the OPD).

## Discussion

The numbers of refugees arriving and being allocated to the city of Cologne started to rise during autumn 2014. In response to health needs exemplified by frequent calls for emergency health services and within a short period of time a new model for immediate health service for refugees was established in a tripartite effort by the Cologne city authority, the GRC and the ASHIP. The urgency for the emergency accommodation OPD was based on the obvious needs and various barriers to access the official German health care system. The intention was to have a low-threshold and easily accessible health facility on a primary care level to offer necessary initial services, and avoid emergencies and unnecessary calls to city emergency services. The OPD was established as an additional service offer taking care of the special early health needs of refugees to simplify the access to the regular health care system which remains the goal. Our evaluation suggests that, these goals were to a large degree met for this facility.

The offer of adult’s and children’s consultation hours in the Cologne emergency accommodation OPD was generally well received by staff and users. Cooperation between the managing NGO, the GRC, private doctors and the local public health department has proven to be useful. The quality of medical care in the OPD was affected by barriers in language, health and medical concepts and the particular burden this patient group carries from reprisals such as persecution, torture, flight and migration [9]. Furthermore, legal circumstances in the FRG created higher barriers for refugees attempting to access medical care [10].

Our observations concur with those of similar refugee clinics in 3 other German cities (Bremen Frankfurt/Main and Munich) [11–13] (Table 2). Women across all countries of origin attended the OPD more frequently than men, which also concurs with observations in similar services in Bremen and Frankfurt/Main. Unfortunately, direct comparison is limited due to structural differences between the services in the three cities [11, 12]. Since the medical records do not provide fully established medical diagnosis, our data cannot be used to determine the degree to which this difference was due to actual poorer health or health seeking behaviour among women. There was a predominance of acute physical complaints for all refugees in Cologne, as in Bremen and Frankfurt/Main [11, 12]. In Cologne and Bremen, respiratory tract complaints accounted for just fewer than 20% of the presented complaints. The frequent occurrence of nonspecific pain symptoms for adults was similar (Cologne: 23.4%; Bremen: 25.4%) [11]. It can be assumed that the following attributes represent trends for the medical care of refugees in the FGR:

- Women are more likely to be reached through a non-compulsory open consultation service.
- For treatment, acute physical complaints dominate, including acute infections of the respiratory tract and nonspecific pain symptoms such as head, abdominal and back pain.

**Table 2:** Comparison of similar clinics for refugees in Germany in 2015

	Cologne	Bremen	Frankfurt/Main	Munich
place	biggest emergency accommodation of the municipality	every accommodation for asylum seekers in Bremen	public health department	Bavaria barracks (central accommodation)
responsible body	GRC ASHIP public health department	public health department	public health department social service	REFUDOCS e.V.
frequency	twice a week	twice a week to daily (central accommodation)	twice a week	daily
speciality	general medicine, paediatrics	general medicine	general medicine, pre- and postnatal treatment	general medicine, paediatrics, gynaecology
financing	social service (med. treatment), GRC (staff)	public health department (staff)	social service (med. treatment), public health department (staff, rooms)	county of upper Bavaria, municipality of Munich

It should be noted that no further specific diagnoses were made because the focus was on low-threshold acute care. In the OPD, there were neither diagnostics performed concerning geographically specific infectious diseases, nor classification of unspecific pain syndromes with regard to psycho-traumatic causes or any type of screening tests. These diagnostics need to be performed within the regular German health care system. Therefore, conclusions on the disease spectrum of examinations in the OPD have to be drawn with caution. Frequent non-specific symptoms of pain, psychological distress, depression, and trauma must be considered in light of the refugees’ backgrounds and migration experiences as well as their culturally distinct understandings of illness [11, 14, 15].

Refugees should be categorized as a vulnerable group rather than a group posing a health threat to a general population, as it has already been pointed out in the report by the German Robert Koch Institute and the Cologne Statement [7, 16]. This can also be supported by the refugee emergency centre OPD experiences in Munich [13]. The described disease spectrum in the Cologne OPD is comparable to that of the local general population [13, 17]. It has to be emphasized again though that the depth of the diagnostic process was deliberately limited in this setting. Screening tests e.g., for latent tuberculosis are not included.

An introduction to the field of migration and health for all employees working in the health care sector with refugees should be provided. Furthermore, psychological and psychosomatic care should be offered to refugees because of possible traumatization by war and flight. Staff involved in the health service perceived a great unmet need for access to mental health services [18]. In the light of this need, the restriction of mental health services for refugees – as stated in §§ 4 and 6 of the Asylum-Seekers’ Benefits Act, by the German social security service should be re-evaluated.

The need for low-threshold medical care for refugees in all municipal facilities in a single city cannot be met by one local OPD. Local OPDs in large municipal refugee shelters must be supplemented by mobile provision of services for other facilities to ensure the same low-threshold access to medical care and the integration into the German regulatory health care system. At the same time, concerns about patient treatment, cost of treatment and social services billing by the local doctors in the surrounding area must be addressed [19]. Health care for migrants requires knowledge of geographically specific diseases, psycho-traumatisation, culturally different concept of illness and sufficient time for translation which is not always possible to provide in a general practise. Further developments will show how the refugees’ integration into the regular system works and how much additional specialized care is required for this group of people at least for a certain period of time.

The ICPC application for evaluation proved useful because a defined diagnosis – e.g., International Classification of Diseases (ICD) – was rarely achieved.

Tracking possible objective information losses in the existing documentation system was limited because the evaluation by the employees and the experiences during the recording were subjective and loss of information from health data already recorded by treating physicians cannot be excluded. Therefore, our observations support the intention to develop and promote a robust, standardized, mobile documentation system for reliable health records and data for health planning for this specific population. One possibility could be a digital recording of all treatment contacts on the spot using laptops or tablets supplemented by a pre-structured health book. Another possibility would be photo documentation using the patient’s mobile phone. In order to avoid additional work expenditures by duplicate documentation, only the master data (e.g., personal data and the result of an initial examination to rule out infectious diseases such as tuberculosis required by law) should also be noted in the health book. The respective treatment contacts can be added as prints from the digital documentation in the consultation hour. The observed loss of information could thus be alleviated by constant on-site documentation, with improved patient data protection. Digital data collection of treatments would simplify health reporting of the group of refugees in the future [20]. For this purpose, an anonymization of the personal data should be integrated into the program for digital recordings. For the meantime, a copy of the most essential documentation should remain with the patient thus protecting sensitive health data and ensuring availability when people move or are relocated.

### Strength and limitations

This study applied a triangulated approach to obtain the evaluation results by reviewing documents, compiling ICPC coded symptoms, observing processes and interviewing care providers. Patients documentation for the months January to April was only available for clients who returned for a follow up visit after May reducing the number of observations which may have impacted the described distribution of symptoms and complaints. Further, the observation is restricted to one emergency refugee accommodation and its tenants which is however the biggest in Cologne.

## Conclusion

Recent refugees in an emergency refugee accommodation centre OPD in Cologne, Germany were found to have mainly acute infectious respiratory diseases and unspecific complaints of pain that were comparable to local general population treated by general physicians. The service was more intensively used by women and elderly persons. Flexible responses to meet health needs of newly arrived refugees are feasible and necessary. Access to the German regular health care system must be improved. The development of a mobile and pre-structured documentation system should be promoted. One possibility is a digital documentation in the OPD supplemented by a uniform health book that stays with the patient. Photo documentation of health care records with patient mobile phones is another possibility. Psychological support for traumatized refugees must be improved. The legal circumstances and the cost-bearing responsibilities must be reconsidered. An introduction to the field of migration health and review of intercultural understandings of health and illness should be offered for the staff members working in OPDs for refugees across the FDG.

## Additional file

Additional file 1: Questionnaire for service assessment. (DOCX 24 kb)

## Abbreviations

ASHIP: Association of Statutory Health Insurance Physicians (German: Kassenärztliche Vereinigung (KV)
ENT: Diseases concerning ears, nose and throat
FRG: Federal Republic of Germany
GRC: German Red Cross
ICPC: International Classification of Primary Care
OPD: Outpatient department/clinic
UNHCR: United Nations High Commissioner for Refugees

## Footnotes

1 We mainly used the term “refugee” for the description of the observed group of people in the study and while providing background information. In Germany, the legal status of a registered refugee becomes “asylum seeker” which is related to the possibility to get social services. Due to this, we used the term “asylum seeker” in the description of the legal background.

2 The description “Western Balkan” includes the countries of Albania, Bosnia, Kosovo, Macedonia, Montenegro, and Serbia. Middle East includes the countries Afghanistan, Bangladesh, Lebanon, India, Iraq, Iran Palestine, and Pakistan. North Africa includes the countries Algeria, Egypt, and Morocco. Africa includes the countries Angola, Congo, Eritrea, Gabon, Ghana, Guinea, Mali, Nigeria, Senegal, Sierra Leona, and Somalia. This information is based on the patients’ explanations and do not correspond to the official terms for the countries.
