# Supplementary material for: Health care provision for refugees in Germany – one-year evaluation of an outpatient clinic in an urban emergency accommodation
Source: BMC Health Serv Res. 2018 Jun 25;18:488. doi: 10.1186/s12913-018-3174-y (PMC6016127; doi:10.1186/s12913-018-3174-y)
Supplement: Supplementary file 1 — Questionnaire for service assessment. (DOCX 24 kb) [file 12913_2018_3174_MOESM1_ESM.docx]

Structured questionnaire for care providers at the OPD for refuges (translated from German)

Personal Information

Name:

First Name:

DoB:

Gender:

Professional qualification:

Tasks in OPD:

Migration history:  Yes  No

Personal work in OPD and motivation

1. How did you know about the OPD project?

health authority

Media

chamber of physicians

job announcement

Friends

others:

2. Since when do you practice in the OPD? (Start date)

3. Up to when did you work there? (end date)

Still ongoing:  end date:

4. How frequent did you work in the OPD so far?

0-5 days  5-10 days  10-20 days  20-40 days  > 40 days

5. Please describe your personal motivation to engage in the OPD work

Internal organisation of the OPD

*Please mark your degree of agreement /disagreement on the scale from 0-10 by moving the X underneath the scale.*

*e.g.: How intense is your pain?*

⎟_________________________________________________⎟

X

No pain at all unbearable

6. How do you rate the OPD infrastructure?

⎟__________________________________________________⎟

Very bad excellent

7. How do you rate the medical equipment?

⎟__________________________________________________⎟

Very poor excellent

8. How do you rate the staffing?

⎟__________________________________________________⎟

Completely understaffed exactly right

Please tick:

9. How do you rate the opening hours?

too many

too few

exactly right

10. How do you judge the documentation?

too extensive

too little

unsystematic

just appropriate

exactly right

11. Based on the existing documentation there is a good exchange of information among care providers.

⎟__________________________________________________⎟

I disagree completely I fully agree

12. Because of the practiced system of documentation already obtained information get lost.

⎟__________________________________________________⎟

I disagree completely I fully agree

Cooperation between health authority and other care providers

13. According to your experience how well did the referral into the regular health system function?

⎟__________________________________________________⎟

Very poor without any difficulty

14.How well does the communication work among providers and clients during the service at the OPD?

⎟__________________________________________________⎟

Very poor without any difficulty

15. How do you rate the communication between the attending doctors work?

⎟__________________________________________________⎟

Very poor excellent

16. How do you rate the communication with the managing organisation GRC?

⎟__________________________________________________⎟

Very poor excellent

17. How do you rate the communication with the municipal health authority?

⎟__________________________________________________⎟

Very poor excellent

18. During your work did you experience at any time difficulties in issuing of health insurance form by the municipal social welfare office?

Yes

No

19. During your work did you experience at any time difficulties in compensation claims based on the health insurance form of the municipal social welfare office?

Yes

No

Patients/ health complaints/ utilization

20. How do you think the OPD service was accepted by the refugee population?

⎟__________________________________________________⎟

Very poor excellent

21. Do you think that certain subgroups preferentially utilize the OPD service?

Yes

No

If yes, which group(s):

22. Do you think that certain subgroups are not reached by the OPD service?

Yes

No

If yes, which group(s):

23. Which kind of difficulty did you face during patient encounters?

Please select the 2 most important ones from your perspective.

language barrier

intercultural problems

traumatization of patients

level of education

current living conditions of the refugees

social situation of the clients

specific and unusual spectrum of health problems / diagnosis

others:

24. Which important difference do you notice in comparison with your usual work in your own clinical practice?

Please select the 2 most important ones from your perspective.

language barrier

intercultural problems

traumatization of patients

level of education

current living conditions of the refugees

social situation of the clients

specific and unusual spectrum of health problems / diagnosis

others:

25. Which similarities/overlaps do you notice in comparison with your usual work in your own clinical practice?

Please select the 2 most important ones from your perspective.

language barrier

intercultural problems

traumatization of patients

level of education

current living conditions of the refugees

social situation of the clients

specific and unusual spectrum of health problems / diagnosis

others:

:

26. According to your perception which are the reasons for clients to attend the OPD service?

Please select the 2 most important ones from your perspective

acute physical complaints/illness

chronic physical problems/ exacerbation or general advice

unspecific

pregnancy

suspected mental health symptoms

need for report compensation

need for medical report/certificate

others:

27. Which are the most frequently encountered medical problems? Please select 2.

cardio-vascular conditions

endocrinological conditions

respiratory tract infections

gastrointestinal complaints

neurological conditions

muscular- skeletal symptoms

skin conditions

others:

Ideas for improvement / criticism / potential for development

28. What is your general view on the services offered in the OPD service?

Please give 2 positive aspects of the OPD service

-

-

Please give 2 negative aspects of the OPD service

-

-

29. Where do you see gaps in and limitations of the current OPD service?

30. Do you have concrete ideas how to improve the service?
